# Organizational Downsizing and Depressive Symptoms in the European Recession: The Experience of Workers in France, Hungary, Sweden and the United Kingdom

**DOI:** 10.1371/journal.pone.0097063

**Published:** 2014-05-19

**Authors:** M. Harvey Brenner, Elena Andreeva, Töres Theorell, Marcel Goldberg, Hugo Westerlund, Constanze Leineweber, Linda L. Magnusson. Hanson, Ellen Imbernon, Sophie Bonnaud

**Affiliations:** 1 Department of Behavioral and Community Health, School of Public Health, University of North Texas Health Science Center, Fort Worth, Texas, United States of America; 2 FG Epidemiologie, Fakultät VII, Technische Universität Berlin, Berlin, Germany; 3 Institute for Stress Research, Stockholm University, Stockholm, Sweden; 4 Inserm, Centre for Research in Epidemiology and Population Health, U1018, Population-based Cohorts Research Platform, F-94807, Villejuif, France; 5 Public Health Department, Université de Versailles Saint Quentin, Villejuif, France; 6 Département Santé Travail, Institut de veille sanitaire, Saint Maurice, France; The University of Edinburgh, United Kingdom

## Abstract

**Background:**

Organizational downsizing has become highly common during the global recession of the late 2000s with severe repercussions on employment. We examine whether the severity of the downsizing process is associated with a greater likelihood of depressive symptoms among displaced workers, internally redeployed workers and lay-off survivors.

**Methods:**

A cross-sectional survey involving telephone interviews was carried out in France, Hungary, Sweden and the United Kingdom. The study analyzes data from 758 workers affected by medium- and large-scale downsizing, using multiple logistic regression.

**Main Results:**

Both unemployment and surviving layoffs were significantly associated with depressive symptoms, as compared to reemployment, but the perceived procedural justice of a socially responsible downsizing process considerably mitigated the odds of symptoms. Perception of high versus low justice was assessed along several downsizing dimensions. In the overall sample, chances to have depressive symptoms were significantly reduced if respondents perceived the process as transparent and understandable, fair and unbiased, well planned and democratic; if they trusted the employer’s veracity and agreed with the necessity for downsizing. The burden of symptoms was significantly greater if the process was perceived to be chaotic. We further tested whether perceived justice differently affects the likelihood of depressive symptoms among distinct groups of workers. Findings were that the odds of symptoms largely followed the same patterns of effects across all groups of workers. Redeploying and supporting surplus employees through the career change process–rather than forcing them to become unemployed–makes a substantial difference as to whether they will suffer from depressive symptoms.

**Conclusions:**

While depressive symptoms affect both unemployed and survivors, a just and socially responsible downsizing process is important for the emotional health of workers.

## Introduction

Organizational downsizing has become highly common during the global recession of the late 2000s, with severe repercussions on employment. Between 2007 and 2009, global unemployment increased by almost 34 million [1]. In economically difficult times, layoff decisions are often driven by the logic of business survival and competitiveness. Yet even when the downturn subsides, downsizing for structural reasons will hardly disappear from the list of strategies aimed at improving the efficiency of firms [2]. It is a continuous situation of business life in which jobs are both created and destroyed. These processes are often intended as an adjustment of companies to changes in the global market. Meanwhile, growing research evidence suggests adverse effects of downsizing on workers’ health [3]. Depression is one of the most common health conditions in the workforce. It is ranked as the leading cause of disability worldwide [4]. Recent studies considered a variety of pathways by which downsizing may increase depression risks. They include changes in the major category of employment status, such as job displacement (i.e., involving the unemployed and reemployed) [5]–[8], specifically, unemployment or underemployment [9]–[13] and surviving layoffs (i.e., remaining at work after organizational downsizing) [14]–[17]. Further risk factors are closely linked with the threat of such changes [18], their stressful consequences [19]–[21] and maladaptive health behaviors used to cope with the stress [14]; [22].

Virtually all research on downsizing and depression has been dominated by the focus on outcomes of the personnel reduction in the post-layoff period. The downsizing process itself has undeservedly received little attention by occupational health researchers. Published papers report descriptive and qualitative results [23]–[26]. These results indicate that workers’ morale and productivity are directly affected by the way layoffs are implemented. The mismanagement of the downsizing process was perceived by workers as a major source of emotional distress and depression. Employees consistently articulated painful feelings stemming from an unfair process, poor communication of layoff plans and lack of involvement in organizational decision-making. Furthermore, the manner in which terminations are handled can apparently be more traumatic than the terminations themselves [27]. However, no attempts have yet been made to analyze these effects. It is unknown whether the subjective perception of the process differs in displaced–i.e., unemployed “victims”, internally redeployed workers or “survivors” who remained at their workplace. We wish to understand which aspects of the process will be significantly associated with depressive symptoms when altered employment status is taken into account.

Employees’ perception of fairness and justice at the workplace is conceptualized in the framework of organizational justice [28]–[30]. Researchers have most widely tested three dimensions of this concept. Procedural justice is fostered when decision making rules followed in organizations are fair. This implies the accuracy of information collected for making decisions, suppression of bias and representativeness of viewpoints from all affected parties [31]. Distributive justice refers to the fairness of decision outcomes and resource allocation [32]. Finally, relational justice describes equity, politeness and fairness in the interpersonal treatment of employees by supervisors [33]. Associations between justice and psychological health have been consistently documented [34]–[37]. Findings from several well-designed prospective studies suggest that depressive symptoms could represent the consequence of perceived injustice [38]–[41]. We contribute to this literature theoretically and empirically, by proposing and testing new measures of organizational justice in the context of downsizing. Specifically, perceptions of procedural justice are recognized as most important to employees during times of large-scale organizational changes [30].

The aim of the present study is to fill the gap in the knowledge concerning relationships between various aspects of the downsizing process and depressive symptoms. We investigated perceptions of affected workers along multiple dimensions: advance notification; communication of impending redundancies; clarity, fairness and transparency of the downsizing process; the degree to which the downsizing is perceived as well planned or chaotic; extent of democracy in decision-making and opportunity to influence the process; impact of personal factors on dismissals; trust in the truthfulness of the employer; and agreement with the necessity of downsizing. In the situation of workers threatened by involuntary unemployment, we studied perceived support in terms of financial compensation, retraining or any other help by employers for smoothing the transition to new employment. These dimensions have been widely discussed in theoretical and policy-oriented papers focused on the idea of responsible restructuring [27]; [42]–[45]. While this idea is in keeping with the organizational justice approach, it is largely concerned with the policies and practices of organizational change, and with planning and managing their strategic aspects in a humane and resource-efficient manner. This focus recognizes that people are the source of innovation and renewal; workers should be treated as assets to be developed rather than costs to be eliminated. Therefore, redundancies should be used by firms as a last resort, when a wide range of measures fail to produce expected financial viability [42]. Yet if layoffs are unavoidable, downsizing in a socially responsible way foresees strategies to mitigate the negative impact on affected workers. The pre-layoff support in the form of retraining programs and early warning has been identified as crucial for the post-layoff adjustment of “victims” [43];[44]. The optimal management of downsizing should involve workers in the process of organizational change, open and honest communication, clear and fair criteria for terminations and assistance to departing employees [43]; [45]. In sum, responsible restructuring should be aimed at implementing the procedural, relational and distributive aspects of organizational justice. The main research hypothesis of our epidemiological study was formulated in advance. We assumed that the downsizing process carried out in a socially responsible way will mitigate the likelihood of depressive symptoms in affected workers.

## Methods

### Ethics Statement

Our cross-sectional survey was carried out in four European countries: France, Hungary, Sweden, and United Kingdom. The national parts of the study have been approved by the respective research ethics committees: in Hungary, the approval has been received from the Scientific Research Ethics Committee of the Medical Research Council, Budapest (Ref.no: TUKEB 187/2008), in Sweden, the Restructuring Survey has been approved by the Regional Research Ethics Committee, Stockholm (Ref.no: 2009/337-32) and London (Kingston University, approval from January 22, 2010). The French part of the survey received approval from the “Comité Consultatif sur le Traitement de l’Information en matière de Recherches dans le domaine de la Santé (CCTIRS)” of the Ministry of Research (March 12, 2009) and the legal authorization of the “Commission Nationale de l’Informatique et des Libertés (CNIL)” on April 23, 2009. All participants provided their written informed consent. They received an invitation letter with information about the study.

### Study Sample

The Restructuring Survey was designed to address some of the major gaps in research on organizational practices for mitigating the negative impact of downsizing on workers’ health. The study settings were chosen according to specified criteria relevant to downsizing and its aftermath for workers. Specifically, there are different models of social protection, health systems and flexibility of labor markets. The conditions shaped by these models are likely to affect flexibility and security of transitions between jobs in cases of downsizing. Hungary, Sweden, France and the UK represent, respectively, the Eastern European, Nordic, Mediterranean-Continental and Anglo-Saxon models. Included in each country were respondents who worked in small, medium and large organizations with 10 or more employees. Data were collected in telephone interviews between April 2009 and mid-May 2011 from two groups of respondents: (1) employees who have never experienced downsizing and (2) workers from recently downsized companies. A downsizing event was considered to be recent if it happened in the last two years preceding the interview. We focused only on larger scale events with cuts in at least 10% of the workforce, because they are less individually selective: general economic requirements are responsible for layoffs, rather than personal characteristics of workers [46]. The downsized group consisted of displaced workers still unemployed or already reemployed at interview, layoff survivors and internally redeployed employees in occupational transitions. We excluded farmers, the self-employed, workers in micro-businesses and those notified of downsizing who retired, quit or found another job before becoming unemployed. Additionally excluded were non-working persons with no experience of downsizing, as well as people who sustained a non-recent or small-scale downsizing.

Given the strict inclusion criteria, we used a targeted selection of respondents from a variety of sources. The Hungarian sample was drawn from the Hungarostudy 2006, a follow-up of the nationwide representative survey Hungarostudy 2002 [47]; [48]. In order to achieve a sufficient number of interviews, the Hungarian research team derived an additional random sample from the public telephone book. Two successive waves of the nationally representative Swedish Longitudinal Occupational Survey of Health [49]–[52], 2008 and 2010, included data on downsizing and thus served as a basis for the selection of the Restructuring Survey participants. In France, employed respondents were recruited through occupational physicians in the regions of Franche-Comté and Lyon and the health screening centers located in Brittany and South-West regions. These centers and physicians provide regular free health screenings for salaried employees. The recruitment of unemployed persons was done through the public employment agencies in the suburbs of Paris and Lyon, as well as by running an advertisement in a free public newspaper. This newspaper with a large circulation nationwide announces vacancies for job seekers. In the UK, the study participants were enrolled at the BT, a large international telecommunications company. At the time of our survey, BT employed some 128 000 full time workers including contractors in over 170 countries. In 2009, the company reported massive losses and restructured the work process in large parts of the business with total expected job cuts up to 30 000 within two years [53].

BT sought to retain its permanent workforce through redeployment and retraining in a “transition center”. The purpose of the transition center is to support the surplus employees through the career change process. The redeployed workers consider a number of options, such as secondment (i.e., temporary assignment) outside BT, or even leaving the company, or finding a new role in BT but doing something totally different from what they have done before. According to the statistics provided by the BT Group health adviser, 86% of cases are resolved in the transition center within 6 months, and 96% of redeployed workers stay in BT. The advantage of using the BT sample was that we could study the health effects of redeployment which, to our knowledge, have not yet been analyzed in the civilian labor force. Employees who have never experienced downsizing were enrolled in the survey from units and business lines not affected by job cuts. The group exposed to downsizing consisted of persons redeployed in the transition center, reemployed workers and the downsizing survivors. The employer had no knowledge of who decided to participate in the survey.

In each country, we planned to include a minimum of 292 participants. This sample size was calculated under the following assumptions: the downsized group will be compared with employees who have never experienced downsizing, and the expected prevalence of symptoms will be 25% and 10%, respectively, in order to obtain results with statistical significance of alpha 0.05 (two-sided) and power 0.90. This sample size was adjusted upward up to a maximum of 400 participants in order to account for potential nonresponse or exclusion. We used prevalence data for major health outcomes from occupational health studies as a guide for these assumptions [54]; [55].

**Figure 1:**
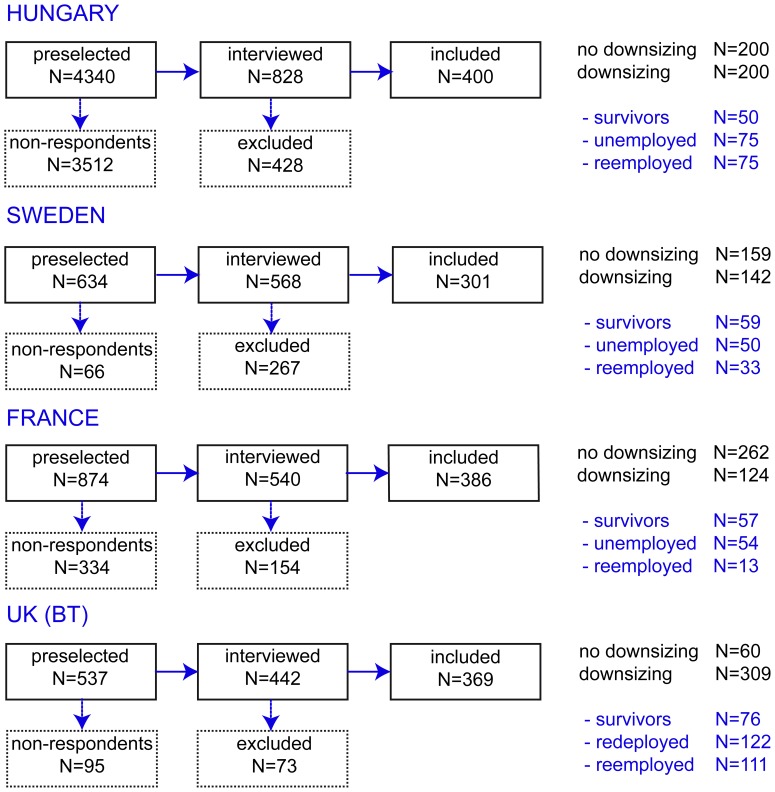
Flow chart showing selection and participation in Restructuring Survey.

Telephone interviews were carried out with preselected persons whose telephone numbers were available. All participants were informed about the aim, scope and duration of the survey and the strict confidentiality in handling personal data. Figure 1 shows how the sample of the Restructuring Survey was created. The participation rates vary by country with nearly 90% for Sweden, 82% for the UK, 62% for France and 19% for Hungary. These rates take into account all refusals and break-offs by respondents, as well as non-interviews due to incorrect telephone numbers, respondents’ never being available or being unavailable during the fieldwork. The lower response rate in the Hungarian sample is caused by the high rate of refusals: nearly 64% of preselected persons declined participation. Interviewers reported a high level of distrust in this population, probably as a result of the socioeconomic situation during the recession of the late 2000s [56]. In all countries, we collected 1456 usable surveys from 681 employees in the non-downsized group and 775 workers exposed to downsizing.

In this report, we analyze data including 758 workers exposed to downsizing with complete responses on all depressive symptoms. Table 1 shows socio-demographic characteristics of these workers and their health habits.

**Table 1:** Characteristics of study participants who experienced downsizing, data are mean (SD) and number (%).

Characteristic	Description	Respondents (N = 758)
Sex	men	452 (59.6%)
	women	306 (40.4%)
Age	years: mean ± SD	46±9.8
Education	university	309 (40.8%)
	any lower education	449 (59.2%)
Country	Hungary	187 (24.7%)
	Sweden	141 (18.6%)
	France	122 (16.1%)
	UK	308 (40.6%)
Employment status	reemployed	225 (29.7%)
	redeployed	121 (16.0%)
	survivor	239 (31.5%)
	unemployed	173 (22.8%)
Smoking	daily or occasional smoker	163 (21.5)
	non-smoker	595 (78.5%)
Frequency of alcohol drinking	“never” (abstainer)	91 (12.0%)
	“once a month or less”	161 (21.2%)
	“2–4 times a month”	208 (27.4%)
	“2–3 times a week”	200 (26.4%)
	“4 times a week or more”	96 (12.7%)
	“don’t know” (non-abstainer)	2 (0.3%)
Sum score for depressive symptoms	mean ± SD	7±5.9

Abbreviations: N (%), number and percent; mean (SD), mean value and standard deviation.

### Questionnaire and Measures

The study questionnaire was designed for use in telephone interviews with a maximum length of 20 minutes. The interview contained two sections: the basic questionnaire which collected demographic and health-related information and the part on downsizing for displaced workers, layoff survivors and redeployed employees.

### Depressive Symptoms

Health-related questions included a brief subscale of depressive symptoms from the Hopkins Symptom Checklist (SCL-90). This subscale has been validated and found to have comparable psychometric properties to longer epidemiological self-report instruments, such as Center for Epidemiological Studies Depression scale (CES-D), but to have superior unidimensionality and consequently more suitable as a dimensional measure of depression severity [57]. It estimates one-week prevalence and consists of six items covering the core symptoms of depression: melancholic mood (“feeling blue”), anhedonia (“feeling no interest in things”), reduced energy and increased fatigability (“feeling lethargy and lack of energy”), excessive worries (“worrying too much”), self-accusation (“blaming yourself for things”) and feeling that “everything is an effort” [58]. Respondents rated how much they have been troubled by each symptom on a five-point Likert scale from “not at all” to “very much”. We computed a sum score ranging from 0 to 24. For the present analysis, the responses were dichotomously categorized (0 = “low level of depressive symptoms”, 1 = ”high level of depressive symptoms”) based on the sum score indicating depression severity. Respondents were regarded to have high level of depressive symptoms if their score exceeded the mean plus the standard deviation of the total Restructuring Survey sample. The clinical significance of high-level symptoms was not further assessed in our respondents. However, the subscale used here was examined before in the Swedish population with reference to the DSM-IV diagnosis of major depression. High-level symptoms (score 17 and above) were predictive of the subsequent antidepressant use and hospitalizations with a depressive episode [57].

### Aspects of the Downsizing Process

The questionnaire section on downsizing was constructed on the basis of relevant scientific literature [27]; [42]; [43]. Downsizing was defined as a process whereby an organization reduces its personnel [46]. In this report, we examined 17 aspects of this process for their potential to affect the likelihood of high-level depressive symptoms. These aspects are listed in Table 2. Unless noted otherwise, workers reported their responses in yes-or-no format. The responses were coded as 1 = yes and 0 = no. First, workers were asked whether they would describe the process of downsizing to be transparent and understandable, fair and unbiased, chaotic or disorganized, well planned and democratic. For this, respondents were requested to think about how decisions were made, no matter if they liked the outcome or not. Second, participants indicated whether they agree that it was necessary to downsize the organization. Third, workers were asked about their influence on downsizing “Did you feel that you could influence how the downsizing was carried out?” and early warning about downsizing “Did your employer give you notification in advance regarding the plans for downsizing?”.

**Table 2:** Responses for experienced downsizing and related conditions, data are number (%).

Aspects of the downsizing process	yes	no	don’t know, refuse	not applicable
Downsizing transparent and understandable	351 (46%)	387 (51%)	20 (<3%)	
Downsizing fair and unbiased	299 (39%)	403 (53%)	56 (7%)	
Downsizing chaotic/disorganized	381 (50%)	353 (47%)	24 (3%)	
Downsizing well planned	298 (39%)	416 (55%)	44 (6%)	
Downsizing democratic	158 (21%)	325 (43%)	43 (6%)	232 (31%)
Agreement with downsizing necessity	359 (47%)	335 (44%)	64 (8%)	
Employee influence on downsizing	81 (11%)	667 (88%)	10 (1%)	
Early warning about downsizing	507 (67%)	249 (33%)	2 (<0.3%)	
Trust in the veracity of employer’s statements	470 (62%)	222 (29%)	66 (9%)	
Influence of personal factors on dismissals	327 (43%)	333 (44%)	98 (13%)	
Manager responsible for staff	188 (25%)	569 (75%)	1 (<0.2%)	
Forced to lay-off personnel	84 (11%)	103 (14%)	1 (<0.2%)	570 (75%)
Financial compensation	149 (20%)	142 (19%)	58 (7%)	409 (54%)
Retraining	194 (26%)	355 (47%)	38 (5%)	171 (22%)
Other help	256 (34%)	293 (39%)	38 (5%)	171 (22%)
Decreased income and benefits after downsizing	220 (29%)	423 (56%)	115 (15%)	
Large-scale downsizing (≥20% laid off)	273 (36%)	229 (30%)	24 (3%)	232 (31%)

Trust in the veracity of the employer’s statements was assessed by the question “Did you think the main reason for downsizing stated by the employer was the true motive?” Interviewees were also queried about the impact of personal factors on dismissals: “Did personal factors influence which employees were dismissed?” The involvement of respondents in laying-off workers was studied in managers responsible for staff. All participants were asked “Were you a manager responsible for staff?” If they answered in the affirmative, they were guided to the question “Were you yourself forced to lay off personnel?” In workers threatened by redundancy, we evaluated support in terms of financial compensation, retraining or any other help by their employers: “Was financial compensation offered in the case of your losing your job?”, “Were you offered re-training to increase your likelihood of getting a new job?”, “Did you receive any other type of help by your company to prepare yourself to find new work?”.

We also included a measure of income loss for all participants who experienced downsizing: “Is your current level of income and benefits: equal to or higher than before the downsizing? … Lower than before the downsizing? … Substantially lower than before the downsizing?” We coded this variable dichotomously indicating 1 for those with decreased income and benefits and 0 for others. Finally, we explored the impact of the downsizing scale: “How large a proportion of the employees were made redundant during downsizing?” The scale included the options “less than 10%”, “between 10% and 20%” and “20% or more.” In workers from recently downsized companies with cuts in at least 10% of the personnel, the variable was coded as 1 for a large-scale downsizing exceeding 20% of staff and 0 for the moderate extent of 10%–20%.

### Employment Status at Interview

We addressed the possibility that the individual outcome of downsizing – employment status at interview – can affect both the perception of the downsizing process and the likelihood of depressive symptoms. Participants from recently downsized companies were asked how they were affected by the downsizing. Workers were classified as displaced if they “were laid off and became unemployed”. This group was further subdivided into those still unemployed or already reemployed at interview, based on yes-or-no responses to the question “Have you got a new job yet?”. The group of layoff survivors consists of workers who stayed in employment in their downsized organizations. Of 239 survivors, 177 (74%) kept their workplaces despite having been notified of possible layoff. In the BT sample, redeployed workers stated that they are “currently in the transition center”. Those who “have been in the transition center for any time during the last two years” but “now have a new job or role which is not a temporary assignment” were classified as reemployed. Employment status is coded as 1 = reemployed (reference group), 2 = redeployed at BT transition center, 3 = layoff survivor and 4 = unemployed at interview. Appendices S1 and S2 show the distribution of responses by employment status for all variables included in the analyses.

### Covariates

Age (in years), gender, education, smoking, alcohol consumption and country-specific effects were examined as potential covariates. Self-reported education included two categories: “university or equivalent degree” versus “any lower education”. For smoking, the dichotomous variable combined daily and occasional smokers versus current nonsmokers. Alcohol consumption was assessed by the question “How often do you drink alcohol?” with the response alternatives coded as 1 = “never” (abstainers, reference group), 2 = “once a month or less”, 3 = “2–4 times a month”, 4 = “2–3 times a week”, 5 = “4 times a week or more”. Four country-specific indicator variables were used to control for unobservable effects, particularly due to national differences in social protection, health systems and flexibility of labor markets. These variables were coded 1 or 0 to denote the country of respondents’ residence. For example, the indicator variable for France equated to 1 in French respondents and 0 in participants from other countries.

### Data Availability

Group level data, statistical code and full details of the explanatory analyses are available from the study research coordinator (elena.andreeva@tu-berlin.de) to all interested researchers upon request, on condition that the European Commission provides its written authorization prior to any distribution of this information. Individual level data of study participants are not available for public data deposition or sharing: the research study is compliant with the ethics and legislation of the data protection in the United Kingdom, France, Hungary and Sweden, according to which necessary arrangements should be met to ensure confidentiality of such data.

### Statistical Analysis

We analyze four sets of questions with multiple logistic regression. First, we examine the relationships between the employment status at interview and depressive symptoms. The rationale for this analysis is based on the previous findings of increased risks of depressive symptoms in unemployed workers [9]–[13] and layoff survivors [14]–[17]. Our study extends the prior literature by including these groups from several countries and adding a new group of internally redeployed civilian workers, which to our knowledge has never been investigated before. Reemployed respondents represent the reference category, since we assumed their status to be most secure and stable in the downsized group, at least in the short term.

Second, we explore the relationships between the employment status at interview and the aspects of the downsizing process. The latter are treated as dependent variables, while employment status is used as a key explanatory variable. We try to shed light on the peculiarities of downsizing in distinct groups of workers. The aim is to examine whether the groups of workers differ with respect to (a) extent of downsizing or income losses, (b) early warning and degree of support in terms of financial compensation, retraining or any other help, and (c) perceptions of the process. Perceptions might vary across groups depending on how closely the individuals were personally affected by downsizing. However, the lack of significant impact of employment status would suggest that perceptions are uniform and unrelated to the current (in)security of employment.

The first two sets of analyses are shown in Table 3. The results are simultaneously adjusted for age, gender, education, smoking, frequency of alcohol drinking and country-specific effects. The measures are modeled as dichotomous variables, except for age (in years), the ordinal frequency of drinking (reference group abstainers) and the categorical “employment status”, which was included in all models as a set of virtual indicator variables.

**Table 3:** Odds ratios and 95% confidence intervals for the associations between employment status, depressive symptoms and dimensions of the downsizing process.

referent: reemployed (OR = 1)		redeployed		survivors		unemployed	
Dependent variables	N	OR (95% CI)	p val.	OR (95% CI)	p val.	OR (95% CI)	p val.
Depressive symptoms	756	1.13 (0.63 to 2.02)	0.684	2.04 (1.26 to 3.31)	0.004	2.85 (1.61 to 5.06)	<0.001
Downsizing transparent, understandable	736	0.75 (0.46 to 1.23)	0.260	1.02 (0.69 to 1.52)	0.906	0.73 (0.46 to 1.15)	0.176
Downsizing fair and unbiased	700	1.06 (0.64 to 1.77)	0.821	1.42 (0.95 to 2.12)	0.086	0.65 (0.40 to 1.06)	0.083
Downsizing chaotic/disorganized	732	1.01 (0.61 to 1.67)	0.961	1.05 (0.71 to 1.56)	0.796	1.12 (0.70 to 1.77)	0.637
Downsizing well planned	712	1.29 (0.77 to 2.16)	0.339	1.01 (0.67 to 1.52)	0.964	1.24 (0.77 to 2.00)	0.376
Downsizing democratic	483	n.a.	n.a.	1.28 (0.70 to 2.33)	0.421	0.89 (0.49 to 1.61)	0.698
Agreement with downsizing necessity	693	1.16 (0.68 to 1.97)	0.581	1.04 (0.69 to 1.58)	0.839	0.52 (0.32 to 0.86)	0.011
Employee influence on downsizing	746	0.93 (0.37 to 2.29)	0.869	1.96 (1.00 to 3.83)	0.050	1.78 (0.80 to 3.99)	0.160
Early warning about downsizing	754	0.89 (0.53 to 1.50)	0.659	2.46 (1.59 to 3.80)	<0.001	1.39 (0.87 to 2.23)	0.169
Trust in the employer’s veracity	690	1.05 (0.61 to 1.82)	0.853	1.27 (0.82 to 1.99)	0.289	0.84 (0.50 to 1.41)	0.516
Influence of personal factors	658	0.93 (0.54 to 1.60)	0.805	0.87 (0.58 to 1.31)	0.515	1.27 (0.79 to 2.06)	0.326
Manager responsible for staff	755	0.93 (0.54 to 1.62)	0.809	1.02 (0.64 to 1.61)	0.940	1.30 (0.75 to 2.26)	0.342
Forced to lay-off personnel	187	0.90 (0.35 to 2.35)	0.835	1.08 (0.48 to 2.45)	0.852	2.35 (0.87 to 6.37)	0.094
Financial compensation	291	n.a.	n.a.	0.66 (0.17 to 2.52)	0.542	1.08 (0.65 to 1.82)	0.772
Retraining	547	0.79 (0.47 to 1.33)	0.372	1.54 (0.60 to 3.99)	0.372	1.18 (0.58 to 2.40)	0.647
Other help	547	1.14 (0.64 to 2.02)	0.655	0.25 (0.10 to 0.62)	0.003	0.87 (0.49 to 1.53)	0.622
Decreased income and benefits	641	1.27 (0.73 to 2.22)	0.404	0.34 (0.21 to 0.55)	<0.001	4.18 (1.99 to 8.77)	<0.001
Large-scale downsizing	502	n.a.	n.a.	0.41 (0.23 to 0.70)	0.001	0.92 (0.55 to 1.55)	0.758

Results from multiple logistic regression analysis.

Variables included in the equations but not shown in the table were age (in years), sex, education, smoking, frequency of alcohol drinking (ordinal variable, referent: abstainer, OR = 1) and country-specific effects.

Abbreviations: N, number of respondents; OR, odds ratio; 95% CI, 95% confidence interval; p val., p value; n.a., not appropriate.

In the third set of analyses, aspects of the downsizing process are treated as key “predictors”. We estimate whether the downsizing process, when carried out in a socially responsible way, mitigates the likelihood of high-level depressive symptoms in affected workers. In Model 1 of Table 4, the adjustment is limited to country-specific effects. Model 2 of Table 4 additionally includes employment status, demographic (age, sex and education) and health-related variables (smoking, frequency of alcohol drinking). Model 3 is further adjusted for decreased income and benefits.

**Table 4:** Odds ratios and 95% confidence intervals for the associations between the dimensions of the downsizing process and depressive symptoms.

Downsizing process	Model 1			Model 2			Model 3		
referent: no (OR = 1)	N	OR (95% CI)	p value	N	OR (95% CI)	p value	N	OR (95% CI)	p value
Downsizing transparent	738	0.60 (0.42 to 0.86)	0.005	736	0.61 (0.42 to 0.88)	0.008	626	0.63 (0.42 to 0.95)	0.026
Downsizing fair and unbiased	702	0.41 (0.28 to 0.60)	<0.001	700	0.40 (0.27 to 0.60)	<0.001	599	0.42 (0.27 to 0.65)	<0.001
Downsizing chaotic	734	2.53 (1.74 to 3.67)	<0.001	732	2.53 (1.73 to 3.69)	<0.001	625	2.79 (1.81 to 4.30)	<0.001
Downsizing well planned	714	0.47 (0.32 to 0.69)	<0.001	712	0.45 (0.30 to 0.67)	<0.001	608	0.40 (0.25 to 0.64)	<0.001
Downsizing democratic	483	0.48 (0.29 to 0.82)	0.006	483	0.50 (0.29 to 0.85)	0.010	380	0.60 (0.33 to 1.08)	0.088
Agreement with downsizing	694	0.51 (0.35 to 0.75)	0.001	693	0.53 (0.36 to 0.79)	0.002	590	0.55 (0.36 to 0.85)	0.007
Employee influence	748	0.78 (0.43 to 1.43)	0.426	746	0.75 (0.41 to 1.39)	0.366	632	0.85 (0.45 to 1.61)	0.617
Early warning	756	0.82 (0.57 to 1.17)	0.274	754	0.76 (0.52 to 1.10)	0.147	641	0.54 (0.36 to 0.82)	0.004
Trust in the employer’s veracity	692	0.49 (0.34 to 0.72)	<0.001	690	0.48 (0.33 to 0.71)	<0.001	589	0.42 (0.27 to 0.65)	<0.001
Influence of personal factors	660	1.50 (1.04 to 2.18)	0.031	658	1.49 (1.02 to 2.17)	0.040	560	1.42 (0.94 to 2.16)	0.099
Manager responsible for staff	757	0.90 (0.60 to 1.35)	0.612	755	0.88 (0.58 to 1.33)	0.543	641	0.89 (0.56 to 1.40)	0.607
Forced to lay-off personnel	187	1.06 (0.52 to 2.16)	0.864	187	1.08 (0.50 to 2.31)	0.843	160	1.47 (0.60 to 3.56)	0.400
Financial compensation	291	0.69 (0.39 to 1.21)	0.196	291	0.60 (0.32 to 1.12)	0.106	183	0.23 (0.08 to 0.64)	0.005
Retraining	549	0.89 (0.55 to 1.44)	0.641	547	0.88 (0.54 to 1.43)	0.595	439	0.80 (0.47 to 1.37)	0.420
Other help	549	0.87 (0.54 to 1.38)	0.545	547	0.91 (0.56 to 1.47)	0.690	437	0.73 (0.41 to 1.28)	0.268
Decreased income/benefits	643	1.40 (0.93 to 2.11)	0.109	641	1.74 (1.12 to 2.71)	0.014		n.a.	n.a.
Large-scale downsizing	502	0.92 (0.60 to 1.42)	0.718	502	0.95 (0.61 to 1.48)	0.806	389	0.86 (0.50 to 1.48)	0.594

Results from multiple logistic regression analysis (dependent variable: depressive symptoms).

Variables included in the equations but not shown in the table were:

Model 1: country-specific effects.

Model 2 = Model 1 plus age (in years), sex, education, smoking, frequency of alcohol drinking (ordinal variable, referent: abstainer, OR = 1) and employment status (referent: reemployed, OR = 1).

Model 3 = Model 2 plus decreased income and benefits (actual responses).

Abbreviations: N, number of respondents; OR, odds ratio; 95% CI, 95% confidence interval; n.a., not appropriate.

Fourth, to examine whether the effects of the downsizing process differently affect the likelihood of high-level symptoms among distinct groups of workers, we re-fitted Models 2 and 3 with interaction terms as Models 2a and 3a, respectively. A summary of these analyses is shown in Appendix S3. The interaction terms were generated between the dichotomously coded dimensions of the downsizing process and employment status (reference group reemployed). We computed odds ratios (ORs) for the impact of downsizing by employment status as point estimates for linear combinations of coefficients after regressions with interaction terms [59];[60]. For each employment status, the odds ratios compare workers who responded to all questions on the dimensions of the downsizing process with “no” (reference, OR = 1) with the persons who answered the respective questions in the affirmative. We used the Wald test to examine whether the interaction terms have a significant contribution in models in addition to the main effects of employment status and the downsizing process [61]. The Wald test explores whether all coefficients of the interaction terms are jointly zero, respectively, OR = 1. For the test statistics, a value of p<0.05 indicates that the interaction terms are jointly significant, consistent with the hypothesis that the impact of the downsizing process on depressive symptoms differs by employment status.

For all analyses, we used Stata/SE 11.2 for Windows. Results are presented in terms of odds ratios (ORs) with 95% confidence intervals (95% CI).

## Results

Our descriptive data in Table 2 show that workers have frequently perceived downsizing as a painful process. Less than half of them felt that it was transparent, understandable, fair, unbiased and well planned. Half of the respondents believed that the process was chaotic. The question about democratic downsizing was omitted in the subsample from the BT transition center due to sensitivities within the business and specific conditions of restructuring in the company: these workers were not involved in the decision making about balancing resources across the business. In other subsamples, more than two-thirds indicated undemocratic downsizing (325 of 483 persons).

Downsizing was experienced by many respondents as an “out of control” process. The vast majority of participants felt they had no influence on how it was carried out. A substantial proportion (44%) disagreed that it was necessary to downsize their organizations. Nearly the same percentage of workers felt that personal factors influenced which employees were dismissed or redeployed. More than one-quarter doubted the veracity of employer’s statements about the main reason for downsizing; an additional 9% were uncertain that their employers revealed their true motives.

Very frequently, workers perceived insufficient support from their employers prior to downsizing. One-third of respondents stated no early warning. Only a minority of Hungarian, French and Swedish participants received retraining or any other help. Such programs were, however, offered to the majority of UK employees in occupational transitions. Nearly 41% of the displaced workers indicated no financial compensation. Many workers experienced decreased income and benefits after the downsizing.

Large-scale downsizing was very common. Massive cuts of ≥20% of the personnel were reported by more than a half of the respondents who were aware of the scale of downsizing in their companies. A question about the proportion of redundancies was omitted in the subsample from the BT transition center due to sensitivities within the organization and a policy of “no compulsory redundancy”.

### Depressive Symptoms, Peculiarities Of Downsizing And Perception Of The Downsizing Process By Employment Status

Table 3 shows associations between the employment status and depressive symptoms. It also presents associations between the employment status and the aspects of the downsizing process. Elevated odds of suffering from a high level of depressive symptoms were observed in persons unemployed at interview (OR = 2.85, p<0.001) and in layoff survivors (OR = 2.04, p = 0.004), as compared to the reemployed group. The BT workers in occupational transitions did not differ from employees with a more stable status with respect to their odds of high-level symptoms.

The results concerning the extent of downsizing and income losses suggest that layoff survivors were less likely to work in organizations with massive job cuts. This is indicated by circa 60% lower odds for the large-scale downsizing in survivors versus reemployed (OR = 0.41, p = 0.001). Remaining in the workplace resulted in lower chances to experience income losses (OR = 0.34, p<0.001). In contrast, unemployment was strongly associated with decreased income and benefits (OR = 4.18, p<0.001).

The group of layoff survivors had a significantly greater probability of early warning (OR = 2.46, p<0.001), but employers were less likely to support survivors with “any other help” for finding a new job (OR = 0.25, p = 0.003). For support in terms of retraining, there were no statistically significant associations with employment status. Similarly, no significant differences were observed in the odds of reporting financial compensation. One group of workers – the BT redeployees in occupational transitions – did not receive financial compensation, because the employer continued paying their salaries.

Regarding perceptions of the downsizing process, employment status showed no statistically significant relationships with trust in the employer’s veracity or influence of personal factors. Furthermore, employment status was not associated with the likelihood of perceiving downsizing as transparent and understandable, chaotic or disorganized, well planned and democratic. However, survivors were more likely to experience the process as fair and unbiased (OR = 1.42, p = 0.086), while this association was reversed in the unemployed (OR = 0.65, p = 0.083); the relationship was almost significant in both groups. Unemployed persons were significantly less likely to agree with the necessity of downsizing in their organizations (OR = 0.52, p = 0.011), as compared to reemployed workers. Layoff survivors were more likely than reemployed to feel that they could influence how the downsizing was carried out; the association was nearly significant (OR = 1.96, p = 0.050).

### Downsizing Process And Perceived Burden Of Depressive Symptoms

Table 4 presents associations between the aspects of the downsizing process and depressive symptoms. After adjustment for country-specific effects, the perception of process as transparent and understandable, fair and unbiased, well planned and democratic was strongly associated with a lower likelihood of depressive symptoms (Model 1). These associations remained significant after additional adjustment for employment status, demographic and health-related variables (Model 2). Odds ratios for reporting high-level depressive symptoms vary in this model from 0.4 for the process perceived as fair and unbiased (p<0.001) to 0.61 for downsizing transparent and understandable (p = 0.008). Workers who perceived downsizing as chaotic had a circa 2.5-fold increase in odds of depressive symptoms (p<0.001).

If workers agreed that it was necessary to downsize their organizations, they had a significantly lower probability of depressive symptoms. In these workers, the odds were reduced approximately twofold (p = 0.002). An even larger reduction was observed for trust in the employer’s veracity (p<0.001). Inversely, the belief of our respondents that personal factors influenced which employees were dismissed or redeployed was strongly associated with a roughly 1.5-fold greater likelihood to score poorly on the scale of depressive symptoms (p = 0.040).

Diminished income and benefits were significantly related to depressive symptoms (OR = 1.74, p = 0.014). However, we encountered a problem of selective item-nonresponse: unemployed participants from Hungary and Sweden had a strong tendency to withhold information about relative changes in their income and benefit levels, despite the interviewers’ assurances of confidentiality. We have therefore checked whether the revealed association is due to limitations in our data; we performed a sensitivity analysis, based on the assumption of decreased income and benefits in the unemployed who failed to provide answers to this question. The results were only marginally affected, with a slight increase in the odds ratio for depressive symptoms (OR = 1.77, p = 0.010).

Displaced workers who were offered financial compensation were less likely to report depressive symptoms. This relationship, however, was statistically non-significant. Small and also insignificant reduction in odds was revealed in respondents who could influence how the downsizing was managed and in those who received a forewarning regarding the employer’s plans for downsizing. We observed no effects of significant magnitude for retraining, other help, managerial position, large-scale downsizing and personal involvement of respondents in laying-off workers.

Estimators were only slightly affected in terms of statistical significance by additional adjustment for decreased income and benefits (Model 3). The associations were somewhat weakened for democratic process (OR = 0.60, p = 0.088) and influence of personal factors (OR = 1.42, p = 0.099) but ameliorated for early warning (OR = 0.54, p = 0.004) and financial compensation (OR = 0.23, p = 0.005). No considerable changes were observed for other variables. The introduction of decreased income and benefits in the model led to a large reduction in the size of our analytic sample due to selective item-nonresponse. We therefore conducted a sensitivity analysis based on the assumption of income loss in all unemployed (not shown). This reversed the majority of changes observed after adjustment for income loss based on actual responses, except for the influence of personal factors, which remained non-significant (OR = 1.42, p = 0.069).

We performed further analyses to test possible interaction effects on depressive symptoms (Appendix S3). The odds ratios in Model 2a indicate some variation in the likelihood of depressive symptoms by employment status depending on the effects of the downsizing process. However, the Wald test shows that the interaction terms are jointly significant only for the early warning (p = 0.013). Early warning appears to decrease the probability of depressive symptoms in the reemployed (OR = 0.68, p = 0.293), redeployed (OR = 0.43, p = 0.053) and layoff survivors (OR = 0.46, p = 0.031), yet the association was reversed in the unemployed respondents (OR = 2.13, p = 0.053). When the results are further adjusted for decreased income and benefits based on the actual responses (Model 3a), there is no evidence of interactions for either dimension of the downsizing process with the employment status. The sensitivity analysis (not shown) assuming income losses in all unemployed revealed jointly significant coefficients of the interaction terms for early warning (p = 0.017 for the Wald test statistics) and elevated nearly significant odds of depressive symptoms in warned unemployed (OR = 2.14, p = 0.053). These results suggest no protective impact of early warning in the unemployed group. For the other dimensions of the downsizing process, odds of depressive symptoms followed the same patterns of effects across employment groups. Thus, odds were reduced if the process was perceived as transparent and understandable, fair and unbiased (significant in survivors and unemployed), well planned (significant in redeployed and survivors) and democratic (mostly significant in unemployed). In contrast, the likelihood of symptoms was elevated in relation to perception of a chaotic process, with the largest significant increase in odds for redeployed and surviving workers. Further comparisons within each status group suggest that our respondents were less likely to suffer from depressive symptoms if they agreed with downsizing necessity. These results were significant in survivors and unemployed. Likewise, trust in the employer’s veracity mitigated odds of symptoms, statistically significant in survivors. Finally, influence of personal factors was linked to high-level symptoms, with significant relationships in the unemployed group.

## Discussion

This is apparently the first multi-country study focusing on a comprehensive assessment of various aspects of downsizing from the standpoint of employees in relation to depressive symptoms. The results indicate that downsizing was frequently perceived as a painful process: non-transparent and poorly understandable, unfair and biased, chaotic or disorganized, poorly planned and undemocratic. Workers have often believed that personal factors influenced dismissals or redeployment. Further, findings demonstrate that the subjective perception of the process is not completely determined by the downsizing outcomes in terms of employment status: the perceptions appear largely uniform and unrelated to the current (in)security of employment. When the process experience is painful, it seems to be emotionally wrenching, and the perceived burden of depressive symptoms is larger. In contrast, workers are less likely to have a higher level of symptoms if the process is experienced as positive. We found a strong association between the perceived influence of personal factors and depressive symptoms in unemployed respondents; this association failed to reach statistical significance in layoff survivors, reemployed and redeployed persons, yet the odds of symptoms followed the same pattern across all employment groups. These results were expected given the earlier findings. In the qualitative research literature, both layoff victims and survivors were outspoken about their bitter feelings if termination decisions appeared arbitrary to them and the process was harsh [27]. Our findings are consistent with the theoretical perspective of procedural justice emphasizing the idea of fair process, fair and consistent decision-making, suppression of bias, accuracy of information and representativeness of the opinions of workers affected [31]. Recent epidemiological studies revealed associations between measures of procedural (in)justice and mental health outcomes among employees, both on a longitudinal basis [36];[38]–[41];[62] and in cross-sectional analysis [35]. However, to our knowledge, this survey is the first to document these relationships with the new measures in the context of the downsizing process.

Another implication of our study is that many workers view the downsizing decisions negatively. There are attitudes of disagreement with the downsizing necessity and distrust in the truthfulness of the employer. Unemployed persons were the least likely to support the idea of downsizing necessity, while trust in the truthfulness of the employer was not significantly related to any of the employment status groups. After adjustment for the employment status and other covariates, the odds for depressive symptoms were reduced more than twofold in workers who trusted their employers regarding the truthfulness of the downsizing reasons stated. A similar effect size was observed in respondents who agreed with the downsizing necessity. These results indicate the importance of the organizational climate during downsizing for the emotional health of workers. The management literature suggests that growing job insecurity due to an accelerated pace of organizational changes can result in a crisis of trust across persons at differential levels of hierarchy in the work setting. This crisis did not exist even a generation ago [63]. Distrust is engendered by opaque decision-making processes [63]. In turn, lack of trust and reciprocity between employers and employees is regarded as a risk factor for depression [64].

Losing a job and remaining unemployed at interview was strongly associated with a reduction in income. Unexpectedly, we observed a strong tendency in unemployed respondents from Hungary and Sweden to deny any information about relative changes in their income and benefit levels. This behavior may be partially explained by the reluctance of the unemployed to raise any issues about “black” (i.e., unreported) income they may earn [56]. Possible concerns about threats to privacy in telephone surveys could also affect their willingness to respond to this question [65]. After adjustment for employment status and covariates, we observed that workers with decreased income and benefits are more likely to score poorly on the scale of depressive symptoms. These findings are well in line with those of prior research: employees with poor financial security are most at risk for poor health during downsizing [66].

Financial compensation was, logically, expected to be inversely associated with depressive symptoms. Our results indicate a tendency towards a lower burden of symptoms in workers who were offered this sort of safety net to temper the income loss inherent in redundancies, but statistical significance was not universally reached. It should however be cautioned that the small numbers of departing employees in our sample (N = 291) could affect the power of these analyses. On an aggregate population basis, the effects of financial compensation may be potentially important. No sizeable associations with depressive symptoms were observed in workers with a managerial position, most specifically, in managers who laid off personnel.

Contrary to expectations based on prior research [22];[66], the scale of downsizing had no association with depressive symptoms. This could be due to a number of factors. First, downsizing scale may not be precisely measured through self-reports of workers. The ideal source would include employer data on redundancies captured at the end of the downsizing event and linked with individual-level data from employees. However, a perfect data source does not exist at present. The earlier studies used employer-based data on downsizing in Finland covering the period of national economic decline in the 1990s. The scale of that decline and the magnitude of job insecurity were almost unequalled in the post-war period, yet job loss was experienced only by employees without permanent contracts [22]. Our data were collected during the global recession of late 2000s, when virtually no job was truly secure. It should also be noted that the earlier studies treated respondents involved in a minor downsizing (<8% of the workforce) as the reference group. Our focus on larger scale events resulted in a more homogeneous sample in terms of respondents being heavily affected by the organizational changes. It is also possible that the global recession was substantially responsible for enlarging the massiveness of downsizing in our sample. Finally, the pervasive nature of recent downsizing might have resulted in increased resilience of workers. There are few publications on this topic, and the hypothesis is not supported by evidence so far: workers showed no increased resilience as they experienced more layoff events [17]. In fact, both employed and non-employed population groups reported more depressed feelings during recession with large-scale layoffs, as indicated by the findings from northern Sweden [67].

In line with much of the recent research, we expected an increased burden of depressive symptoms among the unemployed victims of downsizing and layoff survivors [5]–[17]. The odds of symptoms associated with unemployment in our work is comparable with the average effect size of OR = 2.0 [10] reported in the review of well-designed prospective studies on job loss and distress or emotional ill health [68]–[71]. Becoming unemployed appeared to more than double the risk of depression in the analysis of panel data from Epidemiologic Catchment Area [11]. Null findings in survivors [72] or young unemployed [69] were by and large less frequent. The empirical evidence showed that layoff victims and survivors are exposed to psychosocial adversities with assumed depression-generating effects. These adversities usually include a sequence of stressful events from anticipation of downsizing, through the layoff itself, to adaptation to the altered circumstances. Publications of the last two decades identified sets of stressors typical for each employment status. The risk profile of layoff survivors is characterized by continuous job insecurity due to repeated rounds of downsizing [18], adverse changes in work characteristics [3];[19];[22] and destabilization of the psychosocial climate at work [23]–[25]. These risks can probably outweigh the advantages associated with the status of layoff survivor. In our study, those who kept their jobs had a greater burden of depressive symptoms despite being the lucky ones – somewhat more influential and better informed about the prospect of downsizing, less affected by income losses and massive job cuts. The risks of harm to unemployed layoff victims are not limited to financial strain, disruption to social role, difficulties of reemployment, loss of social support and damage to self-esteem [9];[73];[74]. Our analysis adds that unemployed victims did not feel well prepared for downsizing and job loss. This is indicated by their lack of agreement with the necessity of downsizing. In fact, poor preparedness for downsizing could result from the abrupt nature of layoff events or from insufficient pre-layoff support.

Our results indicate that the strategy of keeping and supporting surplus employees through the career change process – rather than forcing them to become unemployed – makes a substantial difference as to whether they will suffer from a high level of depressive symptoms. In the case of redeployed BT workers, we found no significant increase in the likelihood of depressive symptoms relative to employees with a more secure status. We can thus assume considerable savings in health costs and a good maintenance of worker capacity if organizations could utilize redeployment policies as exemplified by the BT. This study is probably the first to analyze the effects of redeployment in the civilian labor force. Our data suggest that redeployed workers are protected from major risks faced by those displaced. Indeed, maintenance of income level was reported by the vast majority of workers who are or were formerly redeployed in the BT transition center. By contrast, income level deteriorated in the majority of workers with the experience of job loss in the population samples from Hungary, Sweden and France. Skill upgrading and other help was indicated much more frequently by the BT redeployees than by displaced workers.

Contrary to our expectations, no statistically significant protective effects on depressive symptoms were found for retraining, other help and employee influence on downsizing [27];[42]–[45];[73]. Early warning showed an inconsistent pattern of relationships. On closer inspection in models with interaction terms, early warning was linked to a decreased likelihood of symptoms in all groups employed at interview, with significant odds ratios in survivors, but no beneficial effects could be confirmed in those unemployed. These variables were hypothesized to denote the pre-layoff support and the involvement of workers in the process of organizational change. It should however be noted that we analyzed the short-term impact of these strategies. Significant beneficial effects may become visible in a longer term context, while the immediate stage of transition to new employment may place a great strain on the affected workers. Despite bringing better opportunities of new employment, the pre-layoff support does not resolve the question of what the future holds. In particular, many workers might be nervous about entering a retraining program; they may lack confidence in their abilities to acquire new skills and compete in the new world of work [44]. The cross-sectional design could therefore make the protective effects of such programs difficult to detect.

Due to the cross-sectional nature of our data, causal inferences cannot be made. Studying causal relationships between the dimensions of the downsizing process and depressive symptoms would require a longitudinal extension to this study. The main aim of the Restructuring Survey was to provide a detailed snapshot of medium- to large-scale downsizing as perceived by workers in different European economies and to check whether some of the downsizing dimensions are the likely determinants of depressive symptoms. A high level of these symptoms does not necessarily reflect the presence of a clinically significant depressive disorder. Focusing on clinically significant illness only would require a larger sample size to have adequate power. Our results should therefore be interpreted in relation to an increased or decreased risk of depressive morbidity [57]. Existing literature suggests that relationships between the nature of the downsizing process and depressive symptoms, observed in this study, might have occurred because the perception of procedural (in)justice or (dis)trust in organizations led to subsequent emotional health problems. However, research on the affective nature of perception can imply reverse causality [75]: poor emotional health can influence the perception of downsizing. Contrasting both theoretical perspectives by, for example, controlling for negative affectivity and emotional ill health prior to downsizing and testing the longitudinal health effects would be the next research step. Understanding the adverse effects of the downsizing process is important for identifying strategies that can mitigate its negative impact on workers’ health and productivity.

The data collection took place during the most severe recession since the Great Depression. Probably, the need of firms to quickly react to emergencies in the economic crisis could have altered the institutional practices of enacting the downsizing procedures, and our study illustrates the perception of these practices in affected workers. How these trends affected the generalizability of our main findings across time is not clear. The observed relationships may be weaker or stronger during periods of relatively low unemployment. Given the pervasiveness of downsizing practices even in stable times, researchers should continue to study the effects of the downsizing dimensions when the economic conditions are significantly improved.

Further limitations of the study include a potential for selection bias. The UK sample of the BT employees is highly selective due to practices of staff retraining and redeployment [76] which are likely to be beneficial for the mental health of employees, as our results show. Furthermore, it is possible that the most disadvantaged workers in Hungary, Sweden and France – for instance, migrants with insufficient language skills or the working poor without telephone lines – could not participate in the telephone interviews. This could cause the strength of the associations observed in our analysis to be attenuated, since these workers are most likely to suffer from harsh layoff processes and their health consequences.

This multi-country study contributes to the identification of a broad spectrum of possible risk factors related to the process of downsizing. Our results underline the importance of just and socially responsible downsizing processes for the emotional health of workers. We find it reasonable to believe that our study sample is representative in terms of variations in downsizing strategies applied in European organizations with medium- to large-scale reduction in personnel. The BT was selected as an example of a company which uses redeployment rather than redundancy for dealing with surplus employees. However, the results presented here are not necessarily valid outside the European Union. They are also not necessarily generalizable to the population of all European workers. Due to the strict selection criteria applied in this study, our attention was focused on labor market participants who could not withdraw from the downsizing situation. It should be kept in mind that workers exposed to downsizing often have a variety of responses: some can choose withdrawal from the situation through early retirement, while others would find another job before becoming unemployed. Therefore, future research should investigate the generalizability of the findings to the groups of workers beyond the scope of this study. In particular, the health effects of downsizing should be clearly differentiated in potentially healthier and better educated workers who find it easier to obtain new employment before the actual job loss, and in older workers for whom it might be extremely difficult to find new jobs.

## Supporting Information

Appendix S1 **Characteristics of study participants (N = 758): distribution of responses by employment status and depressive symptoms.**
(DOC)

Appendix S2 **Aspects of the downsizing process: distribution of responses by employment status and depressive symptoms, data are number (percent).**
(DOC)

Appendix S3 **Odds ratios and 95% confidence intervals for the associations between the dimensions of the downsizing process and depressive symptoms, by employment status.**
(DOC)
