# Supplementary material for: Organizational Downsizing and Depressive Symptoms in the European Recession: The Experience of Workers in France, Hungary, Sweden and the United Kingdom
Source: PLoS One. 2014 May 19;9(5):e97063. doi: 10.1371/journal.pone.0097063 (PMC4026141; doi:10.1371/journal.pone.0097063)
Supplement: Appendix S1 — (DOC) [file pone.0097063.s001.doc]

**Appendix S1** Characteristics of study participants (N=758): distribution of responses by employment status and depressive symptoms

| **Characteristic** | **Employment status** | | | | | **Depressive symptoms (level)** | | |
| --- | --- | --- | --- | --- | --- | --- | --- | --- |
|  | **reemployed** | **redeployed** | **survivors** | **unemployed** | **p value** | **high** | **low** | **p value** |
| Sex |  |  |  |  | 0.058 † |  |  | 0.150 † |
| men | 131 (17.3%) | 85 (11.2%) | 141 (18.6%) | 95 (12.5%) |  | 95 (12.5%) | 357 (47.1%) |  |
| women | 94 (12.4%) | 36 (4.8%) | 98 (12.9%) | 78 (10.3%) |  | 78 (10.3%) | 228 (30.1%) |  |
| Age: years, mean ± SD | 45.8 ± 9.9 | 50.7 ± 5.7 | 45.9 ± 9.8 | 42.9 ± 10.7 | <0.001 ‡ | 46.1 ± 8.8 | 45.9 ± 10.1 | 0.780 ‡ |
| Education |  |  |  |  | 0.018 † |  |  | 0.540 † |
| university | 82 (10.8%) | 41 (5.4%) | 116 (15.3%) | 70 (9.2%) |  | 74 (9.8%) | 235 (31.0%) |  |
| any lower education | 143 (18.9%) | 80 (10.6%) | 123 (16.2%) | 103 (13.6%) |  | 99 (13.1%) | 350 (46.1%) |  |
| Country |  |  |  |  | <0.001 † |  |  | 0.011 † |
| Hungary | 68 (9.0%) |  | 47 (6.2%) | 72 (9.5%) |  | 48 (6.3%) | 139 (18.4%) |  |
| Sweden | 33 (4.3%) |  | 59 (7.8%) | 49 (6.5%) |  | 18 (2.4%) | 123 (16.2%) |  |
| France | 13 (1.7%) |  | 57 (7.5%) | 52 (6.9%) |  | 26 (3.4%) | 96 (12.7%) |  |
| UK | 111 (14.6%) | 121 (16.0%) | 76 (10.0%) |  |  | 81 (10.6%) | 227 (30%) |  |
| Employment status |  |  |  |  |  |  |  | 0.122 † |
| reemployed |  |  |  |  |  | 39 (5.2%) | 186 (24.5%) |  |
| redeployed |  |  |  |  |  | 29 (3.8%) | 92 (12.2%) |  |
| survivor |  |  |  |  |  | 59 (7.8%) | 180 (23.7%) |  |
| unemployed |  |  |  |  |  | 46 (6.1%) | 127 (16.7%) |  |
| Smoking |  |  |  |  | <0.001 † |  |  | 0.705 † |
| daily or occasional smoker | 44 (5.8%) | 8 (1.1%) | 45 (5.9%) | 66 (8.7%) |  | 39 (5.1%) | 124 (16.4%) |  |
| non-smoker | 181 (23.9%) | 113 (14.9%) | 194 (25.6%) | 107 (14.1%) |  | 134 (17.7%) | 461 (60.8%) |  |
| Frequency of alcohol drinking |  |  |  |  | <0.001 † |  |  | 0.249 † |
| “never” (abstainer) | 33 (4.4%) | 16 (2.1%) | 19 (2.5%) | 23 (3.0%) |  | 26 (3.4%) | 65 (8.6%) |  |
| “once a month or less” | 47 (6.2%) | 11 (1.5%) | 55 (7.3%) | 48 (6.3%) |  | 35 (4.6%) | 126 (16.6%) |  |
| “2-4 times a month” | 62 (8.2%) | 23 (3.0%) | 72 (9.5%) | 51 (6.7%) |  | 38 (5.0%) | 170 (22.4%) |  |
| “2-3 times a week” | 53 (7.0%) | 46 (6.1%) | 67 (8.8%) | 34 (4.5%) |  | 46 (6.1%) | 154 (20.3%) |  |
| “4 times a week or more” | 28 (3.7%) | 25 (3.3%) | 26 (3.4%) | 17 (2.2%) |  | 27 (3.6%) | 69 (9.1%) |  |
| “don’t know” (non-abstainer) | 2 (0.3%) |  |  |  |  | 1 (0.1%) | 1 (0.1%) |  |
| Sum score (DS): mean ± SD | 6 ± 5.9 | 6.9 ± 5.7 | 7.5 ± 6 | 7.7 ± 5.8 | 0.013 ‡ | 15.9 ± 3.3 | 4.4 ± 3.5 | <0.001 ‡ |

Abbreviation: N (%), number and percent; mean (SD), mean value and standard deviation; sum score (DS), Sum score for depressive symptoms

† p values for Pearson’s χ2 test of between-group differences

‡ p values for the analysis of variance of between-group differences
