# Supplementary material for: Organizational Downsizing and Depressive Symptoms in the European Recession: The Experience of Workers in France, Hungary, Sweden and the United Kingdom
Source: PLoS One. 2014 May 19;9(5):e97063. doi: 10.1371/journal.pone.0097063 (PMC4026141; doi:10.1371/journal.pone.0097063)
Supplement: Appendix S2 — (DOC) [file pone.0097063.s002.doc]

**Appendix S2** Aspects of the downsizing process: distribution of responses by employment status and depressive symptoms, data are number (percent)

| **Aspects of the downsizing process** | | **Employment status** | | | | | **Depressive symptoms (level)** | | |
| --- | --- | --- | --- | --- | --- | --- | --- | --- | --- |
|  |  | **reemployed** | **redeployed** | **survivors** | **unemployed** | **p val.** | **high** | **low** | **p val.** |
| Downsizing transparent |  |  |  |  |  | 0.210 |  |  | 0.003 |
|  | no | 107 (14.5%) | 70 (9.5%) | 114 (15.4%) | 96 (13.0%) |  | 105 (14.2%) | 282 (38.2%) |  |
|  | yes | 109 (14.8%) | 50 (6.8) | 118 (16.0%) | 74 (10.0%) |  | 63 (8.5%) | 288 (39.0%) |  |
| Downsizing fair and unbiased |  |  |  |  |  | 0.004 |  |  | <0.001 |
|  | no | 120 (17.1) | 63 (9.0%) | 111 (15.8%) | 109 (15.5%) |  | 122 (17.4%) | 281 (40.0%) |  |
|  | yes | 88 (12.5%) | 49 (7.0%) | 112 (16.0%) | 50 (7.1%) |  | 45 (6.4%) | 254 (36.2%) |  |
| Downsizing chaotic |  |  |  |  |  | 0.765 |  |  | <0.001 |
|  | no | 106 (14.4%) | 51 (7.0%) | 114 (15.5%) | 82 (11.2%) |  | 52 (7.1%) | 301 (41.0%) |  |
|  | yes | 111 (15.1%) | 66 (9.0%) | 120 (16.4%) | 84 (11.4%) |  | 117 (15.9%) | 264 (36.0%) |  |
| Downsizing well planned |  |  |  |  |  | 0.140 |  |  | <0.001 |
|  | no | 128 (17.9%) | 72 (10.1%) | 135 (18.9%) | 81 (11.3%) |  | 116 (16.3%) | 300 (42.0%) |  |
|  | yes | 83 (11.6%) | 45 (6.3%) | 90 (12.6%) | 80 (11.2%) |  | 46 (6.4%) | 252 (35.3%) |  |
| Downsizing democratic |  |  |  |  |  | 0.147 |  |  | 0.002 |
|  | no | 70 (14.5%) | n.a. | 140 (29.0%) | 115 (23.8%) |  | 88 (18.2%) | 237 (49.1%) |  |
|  | yes | 29 (6.0%) | n.a. | 83 (17.2%) | 46 (9.5%) |  | 23 (4.7%) | 135 (28.0%) |  |
| Agreement with downsizing |  |  |  |  |  | <0.001 |  |  | 0.001 |
|  | no | 90 (12.9%) | 40 (5.8%) | 99 (14.3%) | 106 (15.3%) |  | 93 (13.4%) | 242 (34.9%) |  |
|  | yes | 109 (15.7%) | 70 (10.1%) | 124 (17.9%) | 56 (8.1%) |  | 61 (8.8%) | 298 (42.9%) |  |
| Employee influence |  |  |  |  |  | 0.016 |  |  | 0.297 |
|  | no | 208 (27.8%) | 109 (14.6%) | 200 (26.7%) | 150 (20.1%) |  | 158 (21.1%) | 509 (68.1%) |  |
|  | yes | 15 (2.0%) | 9 (1.2%) | 36 (4.8%) | 21 (2.8%) |  | 15 (2.0%) | 66 (8.8%) |  |
| Early warning about downsizing |  |  |  |  |  | <0.001 |  |  | 0.196 |
|  | no | 91 (12.0%) | 43 (5.7%) | 51 (6.8%) | 64 (8.5%) |  | 64 (8.5%) | 185 (24.5%) |  |
|  | yes | 132 (17.5%) | 78 (10.3%) | 188 (24.9%) | 109 (14.4%) |  | 109 (14.4%) | 398 (52.6%) |  |
| Trust in the employer’s veracity |  |  |  |  |  | 0.247 |  |  | <0.001 |
|  | no | 66 (9.5%) | 37 (5.4%) | 62 (9.0%) | 57 (8.2%) |  | 70 (10.1%) | 152 (22.0%) |  |
|  | yes | 130 (18.8%) | 71 (10.3%) | 167 (24.1%) | 102 (14.7%) |  | 84 (12.1%) | 386 (55.8%) |  |
| Influence of personal factors |  |  |  |  |  | 0.141 |  |  | 0.019 |
|  | no | 101 (15.3%) | 50 (7.6%) | 116 (17.6%) | 66 (10.0%) |  | 65 (9.9%) | 268 (40.6%) |  |
|  | yes | 104 (15.8%) | 43 (6.5%) | 94 (14.2%) | 86 (13.0%) |  | 89 (13.4%) | 238 (36.1%) |  |
| Manager responsible for staff |  |  |  |  |  | 0.791 |  |  | 0.694 |
|  | no | 172 (22.7%) | 87 (11.5%) | 179 (23.7%) | 131 (17.3%) |  | 132 (17.4%) | 437 (57.7%) |  |
|  | yes | 52 (6.9%) | 34 (4.5%) | 60 (7.9%) | 42 (5.5%) |  | 41 (5.4%) | 147 (19.4%) |  |
| Forced to lay-off personnel |  |  |  |  |  | 0.720 |  |  | 0.836 |
|  | no | 30 (16.0%) | 17 (9.1%) | 35 (18.7%) | 21 (11.2%) |  | 22 (11.8%) | 81 (43.3%) |  |
|  | yes | 21 (11.2%) | 17 (9.1%) | 25 (13.4%) | 21 (11.2%) |  | 19 (10.2%) | 65 (34.7%) |  |
| Financial compensation |  |  |  |  |  | 0.704 |  |  | 0.286 |
|  | no | 55 (18.9%) | n.a. | 7 (2.4%) | 80 (27.5%) |  | 35 (12.0%) | 107 (36.8) |  |
|  | yes | 52 (17.9%) | n.a. | 6 (2.1%) | 91 (31.3%) |  | 29 (10%) | 120 (41.2%) |  |
| Retraining |  |  |  |  |  | <0.001 |  |  | 0.712 |
|  | no | 135 (24.6%) | 56 (10.2%) | 32 (5.8%) | 132 (24.0%) |  | 78 (14.2%) | 277 (50.5%) |  |
|  | yes | 81 (14.8%) | 65 (11.8%) | 11 (2.0%) | 37 (6.7%) |  | 40 (7.3%) | 154 (28.0%) |  |
| Other help |  |  |  |  |  | <0.001 |  |  | 0.402 |
|  | no | 104 (18.9%) | 34 (6.2%) | 35 (6.4%) | 120 (21.9%) |  | 67 (12.2%) | 226 (41.2%) |  |
|  | yes | 110 (20.0%) | 87 (15.8%) | 8 (1.5%) | 51 (9.3%) |  | 51 (9.3%) | 205 (37.3%) |  |
| Decreased income / benefits |  |  |  |  |  | <0.001 |  |  | 0.298 |
|  | no | 133 (20.7%) | 85 (13.2%) | 187 (29.1%) | 18 (2.8%) |  | 85 (13.2%) | 338 (52.6%) |  |
|  | yes | 81 (12.6%) | 36 (5.6%) | 48 (7.5%) | 55 (8.5%) |  | 52 (8.1%) | 168 (26.1%) |  |
| Large-scale downsizing |  |  |  |  |  | <0.001 |  |  | 0.285 |
|  | no | 41 (8.2%) | n.a. | 124 (24.7%) | 64 (12.8%) |  | 57 (11.4%) | 172 (34.2%) |  |
|  | yes | 69 (13.7%) | n.a. | 98 (19.5%) | 106 (21.1%) |  | 57 (11.4%) | 216 (43.0%) |  |

Abbreviation: n.a., not appropriate; p. val., p values for Pearson’s χ2 test of between-group differences
