# Supplementary material for: Organizational Downsizing and Depressive Symptoms in the European Recession: The Experience of Workers in France, Hungary, Sweden and the United Kingdom
Source: PLoS One. 2014 May 19;9(5):e97063. doi: 10.1371/journal.pone.0097063 (PMC4026141; doi:10.1371/journal.pone.0097063)
Supplement: Appendix S3 — (DOC) [file pone.0097063.s003.doc]

**Appendix S3** Odds ratios and 95% confidence intervals for the associations between the dimensions of the downsizing process and depressive symptoms, by employment status (continues on the next page)

| **Downsizing process** | **Model 2a** | | | | | **Model 3a** | | | | | |
| --- | --- | --- | --- | --- | --- | --- | --- | --- | --- | --- | --- |
| referent: no (OR=1) | **N** | **OR (95% CI)** | **p val.** | **Wald test*** | | **N** | **OR (95% CI)** | **p val.** | **Wald test*** | | |
|  |  |  |  | **chi2** | **p val.** |  |  |  | **chi2** | | **p val.** |
| Downsizing transparent |  |  |  | 1.06 | 0.787 |  |  |  | 3.09 | | 0.378 |
| reemployed | 214 | 0.80 (0.39 to 1.64) | 0.540 |  |  | 206 | 0.89 (0.43 to 1.84) | 0.763 |  | |  |
| redeployed | 120 | 0.44 (0.18 to 1.10) | 0.080 |  |  | 120 | 0.49 (0.19 to 1.22) | 0.125 |  | |  |
| survivors | 232 | 0.61 (0.32 to 1.14) | 0.119 |  |  | 228 | 0.67 (0.35 to 1.26) | 0.213 |  | |  |
| unemployed | 170 | 0.57 (0.28 to 1.19) | 0.137 |  |  | 72 | 0.14 (0.02 to 1.21) | 0.074 |  | |  |
| Downsizing fair and unbiased |  |  |  | 2.35 | 0.503 |  |  |  | 2.53 | | 0.470 |
| reemployed | 206 | 0.66 (0.31 to 1.43) | 0.291 |  |  | 197 | 0.67 (0.31 to 1.46) | 0.314 |  | |  |
| redeployed | 112 | 0.40 (0.16 to 1.02) | 0.055 |  |  | 112 | 0.44 (0.17 to 1.13) | 0.089 |  | |  |
| survivors | 223 | 0.30 (0.15 to 0.59) | <0.001 |  |  | 220 | 0.33 (0.17 to 0.65) | 0.001 |  | |  |
| unemployed | 159 | 0.37 (0.16 to 0.89) | 0.026 |  |  | 70 | 0.17 (0.02 to 1.50) | 0.111 |  | |  |
| Downsizing chaotic |  |  |  | 7.00 | 0.072 |  |  |  | 6.96 | | 0.073 |
| reemployed | 215 | 1.37 (0.65 to 2.88) | 0.401 |  |  | 206 | 1.21 (0.57 to 2.56) | 0.617 |  | |  |
| redeployed | 117 | 5.20 (1.82 to 14.91) | 0.002 |  |  | 117 | 5.10 (1.78 to 14.65) | 0.002 |  | |  |
| survivors | 234 | 3.89 (1.97 to 7.67) | <0.001 |  |  | 230 | 3.54 (1.79 to 7.02) | <0.001 |  | |  |
| unemployed | 166 | 1.74 (0.84 to 3.57) | 0.134 |  |  | 72 | 5.01 (0.98 to 25.70) | 0.053 |  | |  |
| Downsizing well planned |  |  |  | 6.87 | 0.076 |  |  |  | 6.46 | | 0.091 |
| reemployed | 209 | 0.73 (0.33 to 1.62) | 0.437 |  |  | 199 | 0.86 (0.38 to 1.93) | 0.710 |  | |  |
| redeployed | 117 | 0.13 (0.04 to 0.45) | 0.001 |  |  | 117 | 0.14 (0.04 to 0.48) | 0.002 |  | |  |
| survivors | 225 | 0.37 (0.18 to 0.75) | 0.006 |  |  | 221 | 0.42 (0.20 to 0.88) | 0.021 |  | |  |
| unemployed | 161 | 0.70 (0.34 to 1.44) | 0.334 |  |  | 71 | 0.26 (0.06 to 1.10) | 0.067 |  | |  |
| Downsizing democratic |  |  |  | 0.67 | 0.716 |  |  |  | 0.83 | | 0.662 |
| reemployed | 99 | 0.47 (0.10 to 2.32) | 0.355 |  |  | 91 | 0.38 (0.07 to 1.90) | 0.236 |  | |  |
| redeployed | n.a. | n.a. | n.a. |  |  | n.a. | n.a. | n.a. |  | |  |
| survivors | 223 | 0.60 (0.30 to 1.20) | 0.147 |  |  | 220 | 0.58 (0.28 to 1.18) | 0.133 |  | |  |
| unemployed | 161 | 0.36 (0.14 to 0.96) | 0.042 |  |  | 69 | 1.00 (0.25 to 4.05) | 0.996 |  | |  |
| Agreement with downsizing |  |  |  | 5.18 | 0.159 |  |  |  | 2.90 | | 0.235 |
| reemployed | 198 | 1.09 (0.49 to 2.44) | 0.827 |  |  | 190 | 1.02 (0.45 to 2.30) | 0.962 |  | |  |
| redeployed | 110 | 0.64 (0.26 to 1.57) | 0.331 |  |  | 110 | 0.68 (0.28 to 1.67) | 0.400 |  | |  |
| survivors | 223 | 0.36 (0.19 to 0.70) | 0.003 |  |  | 219 | 0.41 (0.21 to 0.81) | 0.010 |  | |  |
| unemployed | 162 | 0.38 (0.16 to 0.91) | 0.030 |  |  | 45‡ | (empty)‡ |  |  | |  |
| Employee influence |  |  |  | 0.61 | 0.895 |  |  |  | 1.23 | | 0.746 |
| reemployed | 221 | 0.37 (0.05 to 2.92) | 0.343 |  |  | 211 | 0.30 (0.04 to 2.45) | 0.263 |  | |  |
| redeployed | 118 | 0.92 (0.18 to 4.73) | 0.920 |  |  | 118 | 1.06 (0.21 to 5.52) | 0.940 |  | |  |
| survivors | 236 | 0.86 (0.36 to 2.06) | 0.736 |  |  | 232 | 0.93 (0.39 to 2.24) | 0.873 |  | |  |
| unemployed | 171 | 0.73 (0.22 to 2.39) | 0.605 |  |  | 71 | 1.20 (0.27 to 5.32) | 0.807 |  | |  |
| Early warning |  |  |  | 10.77 | 0.013 |  |  |  | 0.80 | | 0.851 |
| reemployed | 221 | 0.68 (0.33 to 1.39) | 0.293 |  |  | 212 | 0.65 (0.32 to 1.35) | 0.251 |  | |  |
| redeployed | 121 | 0.43 (0.18 to 1.01) | 0.053 |  |  | 121 | 0.46 (0.20 to 1.11) | 0.083 |  | |  |
| survivors | 239 | 0.46 (0.23 to 0.93) | 0.031 |  |  | 235 | 0.46 (0.22 to 0.93) | 0.030 |  | |  |
| unemployed | 173 | 2.13 (0.99 to 4.58) | 0.053 |  |  | 73 | 0.72 (0.19 to 2.68) | 0.626 |  | |  |
| Trust in the employer’s veracity |  |  |  | 3.60 | 0.308 |  |  |  | 2.06 | | 0.561 |
| reemployed | 194 | 0.72 (0.32 to 1.60) | 0.419 |  |  | 187 | 0.68 (0.30 to 1.55) | 0.360 |  | |  |
| redeployed | 108 | 0.41 (0.16 to 1.05 | 0.063 |  |  | 108 | 0.43 (0.17 to 1.09) | 0.074 |  | |  |
| survivors | 229 | 0.31 (0.16 to 0.60) | 0.001 |  |  | 225 | 0.32 (0.16 to 0.63) | 0.001 |  | |  |
| unemployed | 159 | 0.66 (0.31 to 1.41) | 0.284 |  |  | 69 | 0.34 (0.08 to 1.55) | 0.164 |  | |  |
| Influence of personal factors |  |  |  | 2.02 | 0.567 |  |  |  | 4.06 | | 0.255 |
| reemployed | 203 | 1.41 (0.68 to 2.94) | 0.359 |  |  | 193 | 1.47 (0.70 to 3.10) | 0.311 |  | |  |
| redeployed | 93 | 1.25 (0.49 to 3.18) | 0.646 |  |  | 93 | 1.17 (0.45 to 3.01) | 0.750 |  | |  |
| survivors | 210 | 1.19 (0.62 to 2.30) | 0.595 |  |  | 206 | 1.10 (0.56 to 2.14) | 0.782 |  | |  |
| unemployed | 152 | 2.43 (1.09 to 5.43) | 0.030 |  |  | 68 | 6.59 (1.27 to 34.26) | 0.025 |  | |  |
| **Downsizing process** | **Model 2a** | | | | | **Model 3a** | | | | | |
| referent: no (OR=1) | **N** | **OR (95% CI)** | **p val.** | **Wald test*** | | **N** | **OR (95% CI)** | **p val.** | | **Wald test*** | |
|  |  |  |  | **chi2** | **p val.** |  |  |  | | **chi2** | **p val.** |
| Manager responsible for staff |  |  |  | 5.99 | 0.112 |  |  |  | | 7.37 | 0.061 |
| reemployed | 222 | 0.43 (0.16 to 1.18) | 0.100 |  |  | 212 | 0.40 (0.14 to 1.11) | 0.078 | |  |  |
| redeployed | 121 | 0.47 (0.16 to 1.35) | 0.161 |  |  | 121 | 0.48 (0.17 to 1.40) | 0.178 | |  |  |
| survivors | 239 | 1.49 (0.76 to 2.92) | 0.246 |  |  | 235 | 1.55 (0.78 to 3.06) | 0.211 | |  |  |
| unemployed | 173 | 1.11 (0.50 to 2.46) | 0.797 |  |  | 73 | 1.94 (0.48 to 7.87) | 0.351 | |  |  |
| Forced to lay-off personnel |  |  |  | 1.79 | 0.617 |  |  |  | | 1.17 | 0.761 |
| reemployed | 51 | 0.74 (0.11 to 5.01) | 0.755 |  |  | 48 | 1.01 (0.14 to 7.29) | 0.994 | |  |  |
| redeployed | 34 | 0.70 (0.10 to 5.05) | 0.727 |  |  | 34 | 0.73 (0.10 to 5.45) | 0.756 | |  |  |
| survivors | 60 | 2.03 (0.61 to 6.77) | 0.247 |  |  | 59 | 2.31 (0.65 to 8.23) | 0.196 | |  |  |
| unemployed | 42 | 0.70 (0.17 to 2.94) | 0.623 |  |  | 19 | 1.39 (0.14 to 14.05) | 0.781 | |  |  |
| Financial compensation |  |  |  | 2.60 | 0.273 |  |  |  | | 0.07 | 0.966 |
| reemployed | 107 | 0.29 (0.08 to 1.04) | 0.058 |  |  | 99 | 0.24 (0.06 to 0.95) | 0.042 | |  |  |
| redeployed | n.a. | n.a. | n.a. |  |  | n.a. | n.a. | n.a. | |  |  |
| survivors | 13 | 0.21 (0.01 to 3.45) | 0.277 |  |  | 12 | 0.30 (0.01 to 6.61) | 0.447 | |  |  |
| unemployed | 171 | 0.83 (0.40 to 1.73) | 0.614 |  |  | 72 | 0.20 (0.04 to 1.00) | 0.051 | |  |  |
| Retraining |  |  |  | 0.96 | 0.811 |  |  |  | | 1.01 | 0.800 |
| reemployed | 214 | 0.82 (0.36 to 1.87) | 0.630 |  |  | 205 | 0.80 (0.35 to 1.86) | 0.609 | |  |  |
| redeployed | 121 | 0.75 (0.32 to 1.75) | 0.513 |  |  | 121 | 0.73 (0.31 to 1.71) | 0.473 | |  |  |
| survivors | 43 | 2.09 (0.30 to 14.66) | 0.458 |  |  | 40 | 2.29 (0.27 to 19.59) | 0.450 | |  |  |
| unemployed | 169 | 0.99 (0.39 to 2.53) | 0.983 |  |  | 73 | 0.71 (0.18 to 2.86) | 0.628 | |  |  |
| Other help |  |  |  | 3.53 | 0.171 |  |  |  | | 2.32 | 0.314 |
| reemployed | 212 | 0.53 (0.24 to 1.19) | 0.124 |  |  | 204 | 0.50 (0.22 to 1.13) | 0.095 | |  |  |
| redeployed | 121 | 1.21 (0.47 to 3.12) | 0.695 |  |  | 121 | 1.14 (0.44 to 2.98) | 0.790 | |  |  |
| survivors | 35† | (empty)† |  |  |  | 32† | (empty)† |  | |  |  |
| unemployed | 171 | 1.50 (0.67 to 3.37) | 0.326 |  |  | 72 | 1.36 (0.30 to 6.20) | 0.688 | |  |  |
| Decreased income / benefits |  |  |  | 1.33 | 0.723 |  |  |  | |  |  |
| reemployed | 212 | 1.35 (0.64 to 2.86) | 0.429 |  |  |  |  |  | |  |  |
| redeployed | 121 | 1.71 (0.70 to 4.15) | 0.236 |  |  |  |  |  | |  |  |
| survivors | 235 | 1.91 (0.92 to 3.99) | 0.084 |  |  |  |  |  | |  |  |
| unemployed | 73 | 4.67 (0.55 to 39.80) | 0.159 |  |  |  |  |  | |  |  |
| Large-scale downsizing |  |  |  | 3.13 | 0.210 |  |  |  | | 3.10 | 0.213 |
| reemployed | 110 | 0.38 (0.12 to 1.16) | 0.090 |  |  | 100 | 0.37 (0.11 to 1.17) | 0.090 | |  |  |
| redeployed | n.a. | n.a. | n.a. |  |  | n.a. | n.a. | n.a. | |  |  |
| survivors | 222 | 1.07 (0.56 to 2.03) | 0.840 |  |  | 218 | 1.00 (0.52 to 1.94) | 0.997 | |  |  |
| unemployed | 170 | 1.20 (0.58 to 2.51) | 0.622 |  |  | 71 | 1.86 (0.35 to 10.00) | 0.469 | |  |  |

Results from multiple logistic regression analysis (dependent variable: depressive symptoms)

Variables included in the equations but not shown in the table were:

Model 2a=Model 2 plus interaction terms (downsizing process x employment status).

Model 3a=Model 3 plus interaction terms (downsizing process x employment status).

Odds ratios for the impact of downsizing by employment status were computed as point estimates for linear combinations of coefficients after regressions with interaction terms.

Wald test* explores whether all coefficients of the interaction terms are jointly zero (respectively, OR=1: no differences in the impact of downsizing process by employment status). A value of p<0.05 indicates that the interaction terms are jointly significant, consistent with the hypothesis that the downsizing process moderates the influence of employment status on risk of depressive symptoms.

Abbreviations: N, number of respondents; OR, odds ratio; 95% CI, 95% confidence interval; n.a., not appropriate

† interaction term dropped from the regression, because being a layoff survivor who received other help perfectly predicted the absence of depression for all 8 observations

‡ interaction term dropped from the regression, because being unemployed who agreed with downsizing necessity perfectly predicted the absence of depression for all 26 observations.
